# Thoracic Endovascular Aortic Repair of Esophageal Cancer-Associated Aortoesophageal Fistula: A Case Report and Literature Review

**DOI:** 10.1155/2018/9851397

**Published:** 2018-12-19

**Authors:** Akiko Sasaki, Hideto Egashira, Shinnosuke Tokoro, Chikamasa Ichita, Satoshi Takizawa, Toshitaka Tsukiyama, Hidemitsu Ogino, Jun Kawachi, Rai Shimoyama, Makoto Kako

**Affiliations:** ^1^Gastroenterology Medicine Center, Shonan Kamakura General Hospital, 1370-1 Okamoto, Kamakura City, Kanagawa 247-8533, Japan; ^2^Intervention Radiology (IVR) and Radiology, Shonan Kamakura General Hospital, 1370-1 Okamoto, Kamakura City, Kanagawa 247-8533, Japan; ^3^General Surgery, Shonan Kamakura General Hospital, 1370-1 Okamoto, Kamakura City, Kanagawa 247-8533, Japan

## Abstract

**Background:**

Thoracic endovascular aortic repair of an aortoesophageal fistula is an effective emergency treatment for patients with T4-esophageal cancer, as it prevents sudden death, and is a bridge to surgery. However, the course of unresectable malignant aortoesophageal fistula treated with thoracic endovascular aortic repair alone is not well-known.

**Case Presentation:**

We report a 67-year-old Japanese man with T4-esophageal cancer who experienced a chemoradiation-induced aortoesophageal fistula and was rescued with thoracic endovascular aortic repair. He recovered after the procedure and survived for 4 additional months with management of a mycotic aneurysm and secondary aortoesophageal fistula with the exposure of the stent graft into the esophagus. Thoracic endovascular aortic repair of aortoesophageal fistula with T4-esophageal cancer extended life for nearly an average of 4 months in the reported cases. As a postoperative complication, the exposure of the stent graft into the esophagus is rare but life-threatening; the esophageal stent insertion was effective.

**Conclusions:**

With postoperative management advances, thoracic endovascular aortic repair can improve survival and increase the quality of life of patients with T4-esophageal cancer.

## 1. Introduction

An aortoesophageal fistula (AEF) is a rare but life-threatening condition that causes upper gastrointestinal tract bleeding. It involves the development of a passage between the aorta and the esophagus after a thoracic aortic aneurysm, ingestion of a foreign body, or advanced esophageal cancer (EC) [1]. Current advances in thoracic endovascular aortic repair (TEVAR) have rendered it the primary recommended method for controlling AEF bleeding. The prognosis of AEF is poor because of rerupturing and/or the development of infections; therefore, esophageal resection with or without esophageal reconstruction, as well as replacement of the descending aorta, should be planned after TEVAR for AEF. However, in some patients with advanced EC, the risk of open surgery for both the aorta and the esophagus is too high given their poor preoperative condition. The outcome after TEVAR without resection of AEF for EC has not yet been demonstrated. Here, we report a case of AEF in a patient with unresectable EC that was treated using TEVAR.

We reported this case at APDW (Asian Pacific Digestive Week) 2016.

## 2. Case Presentation

**Figure 1:**
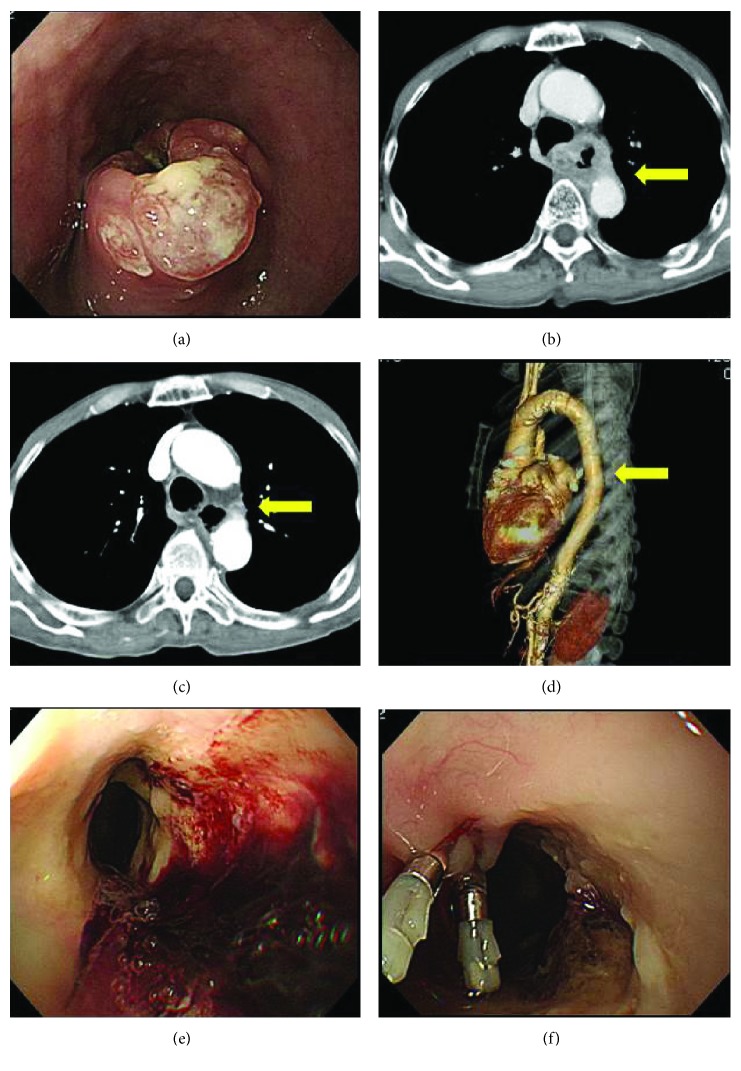
Imaging at the initial treatment for the aortoesophageal fistula using the TEVAR method. (a) EGD revealed a type 2 lesion spanning 3/4 of the circumference of the middle thoracic esophagus. (b) Computed tomography scan (CT) revealed esophageal wall thickening with tumor, and there was loss of the normal fat plane between the esophagus and the adjacent aorta. Approximately 90° of the circumference of the aorta was in contact with the tumor, which suggested aortic invasion (yellow thin arrow). (c) CT scan revealed aortic erosion of the intravenous contrast material within the descending thoracic aorta and extraluminal foci of air between the adjacent esophagus and the aorta (yellow thin arrow). (d) CT angiography did not indicate active bleeding. (e) Emergency esophagogastroduodenoscopy showed a pale fragile esophageal lesion on the posterior wall, an area previously treated with chemoradiation therapy, with massive blood coagulation. (f) Marking clips were placed on the side opposite the lesion.

**Figure 2:**
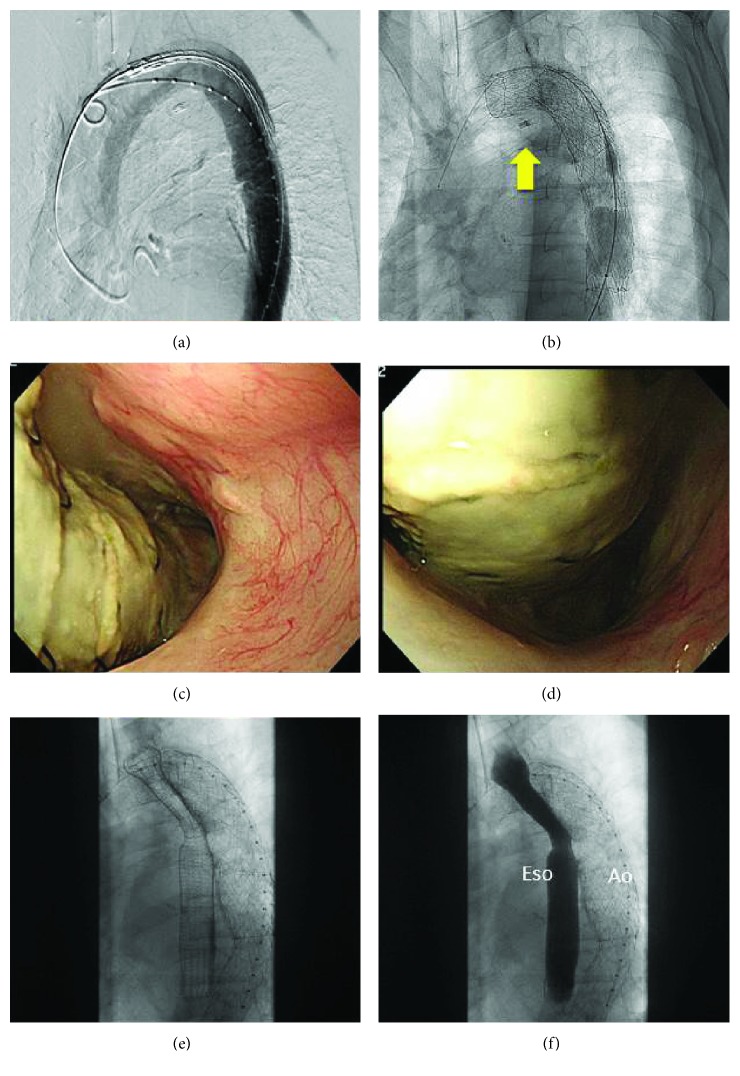
Imaging showed esophageal stenting to repair the aortic stent migration. (a) Aortography showed no active bleeding. (b) Using the marking clips as a reference point, a stent graft was inserted to control the massive esophageal bleeding (yellow bold arrow). (c) Esophagogastroduodenoscopy revealed that the aortic stent is exposed into the esophagus. (d) The stent narrowed the esophageal lumen, hindering the scope's passage past the lesion. (e) An esophageal stent was placed adjacent to the aortic stent to push it out and dilate the esophageal lumen. (f) The contrast medium flowed smoothly within the esophageal stent.

A 67-year-old previously healthy Japanese man initially presented with throat discomfort. He underwent an esophagogastroduodenoscopy (EGD) that revealed a type 2 lesion spanning 3/4 of the circumference of the middle thoracic esophagus (Figures 1(a) and 1(b)). Histopathological examination identified it as squamous cell carcinoma. He was diagnosed with stage III EC (T4N1M0) according to the Union for International Cancer Control tumor-node-metastasis (TNM) system and was treated with chemoradiation therapy (CRT) including fluorouracil and cisplatin plus a radiation dose of 59.4 Gy. On the day of administration of the second cycle of chemotherapy, he had hematemesis and anemia. Upon examination, the patient was hypotensive with a blood pressure of 64/39 mmHg and had tachycardia with an irregular heart rate of 70–120/min. He had a high fever (39.4°C) during the previous 1–2 days with general malaise. Laboratory data revealed a hemoglobin level of 3.9 g/dL. A digital rectal examination revealed diarrhea with fresh blood. After resuscitation with 2.5 L of normal saline administered intravenously, and transfusion with 14 units (1820 mL) of packed red blood cells and 4 units (480 mL) of fresh-frozen plasma, AEF was considered and a computed tomography scan (CT) angiogram of the chest and abdomen was obtained (Figures 1(c) and 1(d)). Although we did not detect active bleeding, the CT scan revealed aortic erosion of the intravenous contrast medium within the descending thoracic aorta, as well as extraluminal foci of air between the adjacent esophagus and the aorta. Emergency EGD showed a pale fragile esophageal lesion on the posterior wall (the area previously treated with CRT), as well as massive blood coagulation in the stomach and duodenum. Marking clips were placed on the side opposite the lesion (Figures 1(e) and 1(f)). Soon after endoscopy, as the patient's condition remained life-threatening, and in the absence of an advance directive from the patient, we performed emergency AEF repair using TEVAR (Figures 2(a) and 2(b)). Under a general anesthetic, TAG® stent grafts (26 mm × 10 cm, 28 mm × 15 cm, and 28 mm × 10 cm) were inserted by vascular surgeons via the right common femoral artery using an 8 Fr sheath. Two TAG stent grafts were deployed from the distal arch, referring to the marking clip. After crimping with the trilobe balloon and confirming the absence of bleeding using a contrast medium, the procedure was completed. The operation time was 1.5 hours. He successfully recovered with stable blood pressure and was discharged 10 days after the TEVAR. Approximately 2 months later, he suffered a mycotic aneurysm around the stent in the ascending aorta. He was treated with meropenem, 1.0 g per day, and vancomycin, 1.0 g per day, for 10 days; cefmetazole, 3.0 g per day, for the following 5 days; and then continued on oral cefuroxime, 750 mg per day. His Eastern Cooperative Oncology Group (ECOG) performance status score decreased from 1 at admission to 3, and he refused further surgery. Three months after the TEVAR, he was readmitted with difficulty in swallowing that began a week prior with worsening of back pain. EGD revealed that the aortic stent was exposed and protruded into the ruptured esophagus (Figures 2(c) and 2(d)). Therefore, an esophageal stent was placed adjacent to the aortic stent to push it out and dilate the esophageal lumen (Figures 2(e) and 2(f)). The patient's back pain improved after the stenting, and he was discharged after 11 days. The patient died 16 days later, approximately 4 months after undergoing emergency TEVAR for AEF.

## 3. Discussion

This report describes the following two points. A TEVAR of AEF with unresectable T4-EC extended the life of the patient for nearly 4 months. As a postoperative complication, the exposure of the stent graft into the esophagus is rare but life-threatening, and esophageal stent insertion was effective. This therapy can effectively control bleeding and hemoptysis, a condition that can be greatly distressing for patients and their families. Even though the combination of TEVAR and esophageal stent cannot provide a long-term survival prognosis, it is still a valuable treatment strategy for patient comfort.

The course of AEF due to EC treated with TEVAR is not well-known. On searching PubMed, we found 916 manuscripts with the search terms “TEVAR” or “Thoracic endovascular aortic repair,” and only 7 of these manuscripts were also identified with the search term “esophageal cancer.”

In a comprehensive 1991 review, Hollander and Quick reported that advanced EC is the third most common etiology of AEF, accounting for 17.0% of cases [1]. CRT is the main therapy for T4 or unresectable EC. The 1- and 3-year survival rates after CRT for those patients were reported to be 41% and 23%, respectively. T4-EC increases the risk of AEF because of the invasion of the aortic walls by cancer cells that are then selectively destroyed with CRT. Deep ulceration and spur formation detected in an upper GI series and a radiation dose over 35 Gy were reported as risk factors for AEF [2].

The operative mortality of open AEF repair, which involves replacement or bypass of the thoracic aorta with concomitant resection or repair of the esophagus, ranges from 45.4% to 55% [3]. TEVAR was originally developed by Dake et al. in 1994 for the treatment of descending thoracic aortic aneurysms. It has a high technical success rate (87.3%) and a more favorable 30-day mortality rate (19.7%), but recurrence of the AEF and stent graft infection is reported in 13.8% and 15.2% of patients who undergo the procedure, respectively [3]. The 30-day mortality rate of TEVAR and surgery is better than that of TEVAR alone (9.4% versus 27.5%, respectively). Considering its limitations, TEVAR for all causes of AEF is considered a bridge therapy to be followed by open repair after the patient is stabilized.

On the other hand, TEVAR alone could be an ultimate treatment for AEF due to T4-EC. T4-EC has a reportedly poor outcome after radical surgery with high morbidity. Thirteen cases of AEFs due to T4-EC treated with TEVAR have been reported, including the present case (Additional file 1) [4–10]. The mean survival was 4.04 ± 3.66 months (range 0.4–59.5 months). One of the 13 patients underwent surgical bypass therapy 2 weeks after TEVAR, while another patient had an esophageal stent inserted 1 week after TEVAR. They were living after more than 6 and 12 months, respectively, after the procedure. There were complications from TEVAR alone, such as 4 cases of infection, including 2 esophagobronchial fistulas and 2 episodes of rebleeding, and 3 cases of secondary aortoesophageal fistulas with the exposure of the stent graft into the esophagus.

There are several interventions reported to prevent complications after TEVAR. Canaud et al. suggested continuing a 6–8-week course of broad-spectrum intravenous antibiotics, followed by lifelong oral antibiotic suppression [3]. There appears to be a protective role for esophageal stent insertion 1 to 2 weeks after TEVAR. Ishikawa et al. reported a successful combined treatment of AEF with TEVAR and an esophageal stent in 2 patients who subsequently achieved 12 and 6 months of survival [9]. In the present case, if stenosis had been present in the esophagus at the time of AEF, it would have been useful to insert a preventive esophageal stent after TEVAR. Ehara et al. recommended CT scans in patients receiving over 30 Gy of CRT in order to select patients who are eligible for preventive TEVAR [2].

In conclusion, TEVAR of AEF with T4-EC extended this patient's life for nearly 4 months. As a postoperative complication, the exposure of the stent graft into the esophagus is rare, and esophageal stent insertion was effective. The proper management after TEVAR alone for T4-EC should be examined, and it may become an essential life-extending tool for T4-EC patients.

## Supplementary Materials

Supplementary Materials Additional file 1: details of thirteen aortoesophageal fistulas cases due to esophageal cancer.
