# Supplementary material for: Thoracic Endovascular Aortic Repair of Esophageal Cancer-Associated Aortoesophageal Fistula: A Case Report and Literature Review
Source: Case Rep Oncol Med. 2018 Dec 19;2018:9851397. doi: 10.1155/2018/9851397 (PMC6313998; doi:10.1155/2018/9851397)
Supplement: Supplementary Materials — Additional file 1: details of thirteen aortoesophageal fistulas cases due to esophageal cancer. [file 9851397.f1.pdf]

Thoracic endovascular aortic repair of esophageal cancer-associated aortoesophageal fistula: a case report and literature review

Akiko Sasaki[1*], Hideto Egashira[1], Shinnosuke Tokoro[1], Chikamasa Ichita[1], Satoshi Takizawa[2], Toshitaka Tsukiyama[2], Hidemitsu Ogino[3], Jun Kawachi[3], Rai Shimoyama[3], and Makoto Kako[1]

[*]**Corresponding author:**

Akiko Sasaki

E-mail: akikomontblanc@yahoo.co.jp

Telephone: +81 467 46 1717, Fax: +81 467 45 0190

Additional file 1 Thirteen cases of aortoesophageal fistulas due to esophageal cancer treated with thoracic endovascular aortic repair

	Year	Age	Sex	path.	Stage	CRT	RTx (Gy)	timing from the initial therapy	Oesophagectomy	esophageal stent	Cause of death	Duration to death from AEF (months)	infection	ABX	rebleeding	stent insertion	others
1	Bergoeing R. M. et al. 2013	62	Male	SCC(node)	IV (T4NXM1)	CRT	51	After	-	-	EC	13	-	+	-	Esophagus	-
2	Dorweiler, B. et al. 2013	60	Female	unknown	IV	unknown	unknown	unknown	unknown	-	EC	7	-	unknown	-	-	-
3	Freezor, R.J. et al. 2009	48	Male	small cell ca	III C(T4N1M0)	-	-	before	-	-	pneumonia due to EP fistula	3	+	-	+	-	Esophagotrachial fistula
4	Ikeda, Y. et al. 2006	64	Male	SCC(mode)	T4NXMX	C for NAC	-	7days after start the course	Bypass: 2w	-	alive	6	-	unknown	-		-
5	Kato, N. et al. 2000	59	Male	SCC(well)	T4NXMX	R	70	During (58/70Gy)	-	-	pneumonia due to EP fistula	4.5	+	+	-	-	Esophagotrachial fistula
6	Ishikawa, N. et al. 2013	75	Male	SCC(mode)	III C(T4N1M0)	CRT	60	1month after finish the course	-	-	sepsis	3	+	-	-	Esophagus	-
7	Ishikawa, N. et al. 2013	81	Male	SCC(mode)	III C(T4N1M0)	R	60	3months after start the course	-	1w after TEVAR	alive	12	-	unknown	-	-	-
8	Okita, Y et al. 2014	64	unknown	unknown	unknown	unknown	unknown	unknown	-	unknown	EC	59.5	unknown	unknown	unknown	unknown	-
9	Okita, Y et al. 2014	65	unknown	unknown	unknown	unknown	unknown	unknown	-	unknown	EC	1	unknown	unknown	unknown	unknown	-
10	Okita, Y et al. 2014	61	unknown	unknown	unknown	unknown	unknown	unknown	-	unknown	EC	2.6	unknown	unknown	unknown	unknown	-
11	Okita, Y et al. 2014	76	unknown	unknown	unknown	unknown	unknown	unknown	-	unknown	EC	0.4	unknown	unknown	unknown	unknown	-
12	Okita, Y et al. 2014	69	unknown	unknown	unknown	unknown	unknown	unknown	-	unknown	EC	1.9	unknown	unknown	unknown	unknown	-
Our case	2013	67	Male	SCC	III C(T4N1M0)	CRT	59.4	during	-	3months after TEVAR	EC	4	+	-	+	Esophagus	-
